# Experimental evidence for the benefits of higher X-ray energies for macromolecular crystallography

**DOI:** 10.1107/S2052252521008423

**Published:** 2021-09-09

**Authors:** Selina L. S. Storm, Danny Axford, Robin L. Owen

**Affiliations:** a Diamond Light Source, Harwell Science and Innovation Campus, Didcot OX11 0DE, United Kingdom

**Keywords:** high-energy X-rays, macromolecular crystallography, X-ray radiation damage

## Abstract

X-ray-induced radiation damage is a limiting factor for the macromolecular crystallographer and data must often be merged from many crystals to yield complete data sets for the structure solution of challenging samples. Increasing the X-ray energy beyond the typical 10–15 keV range promises to provide an extension of crystal lifetime via an increase in diffraction efficiency. To date, however, hardware limitations have negated any possible gains. Through the first use of a cadmium telluride EIGER2 detector and a beamline optimized for high-energy data collection, it is shown that at higher energies fewer crystals will be required to obtain complete data, as the diffracted intensity per unit dose increases by a factor of more than two between 12.4 and 25 keV. Additionally, these higher energy data can provide more information, as shown by a systematic increase in the high-resolution cutoff of the data collected. Taken together, these gains point to a high-energy future for synchrotron-based macromolecular crystallography.

## Abstract 2

Higher X-ray energies are experimentally demonstrated to be highly beneficial for macromolecular crystallography.

## 1. Introduction   

Synchrotron-based macromolecular crystallography (MX) is the method of choice for determining the atomic structures of proteins and viruses, providing almost 90% of Protein Data Bank depositions over the last five years (Goodsell *et al.*, 2020 ▸). More than 95% of these synchrotron-derived depositions were collected using X-rays with energies in the range 10–15 keV (1.240–0.827 Å), reflecting the optimization of sources, beamlines and detectors within this narrow region and, on the sample side, the success of selenomethionine incorporation for experimental phasing at 12.67 keV (Hendrickson *et al.*, 1990 ▸). The continual development of synchrotron beamlines and sources has resulted in the real­ization of smaller beam sizes and increased flux densities at the sample position (Owen *et al.*, 2016 ▸). While these brighter beams enable structure solution from ever-smaller and more challenging crystals, it is at the expense of the one-crystal one-structure approach, as X-ray-induced damage precludes the collection of a complete data set from a single crystal (Smith *et al.*, 2012 ▸). In such cases, the formation of a complete data set is achieved using a multi-crystal methodology, distributing the total dose required for structure solution over many crystals (Liu *et al.*, 2011 ▸; Yamamoto *et al.*, 2017 ▸).

Robust approaches for both collecting and processing multi-crystal data have been developed (Liu *et al.*, 2011 ▸; Giordano *et al.*, 2012 ▸; Foadi *et al.*, 2013 ▸; Zander *et al.*, 2015 ▸; Santoni *et al.*, 2017 ▸; Yamashita *et al.*, 2018 ▸), with the logical endpoint being serial synchrotron crystallography, in which a single diffraction image is collected from each crystal (Diederichs & Wang, 2017 ▸). Rather than collecting from ever more crystals however, a primary aim of a multi-crystal experiment should be to optimize the last experimental step, maximizing the volume of data that can be collected from each crystal, thus reducing sample consumption and simplifying data collection and subsequent analysis.

Increasing the energy of the incident X-rays as a solution to the multi-crystal challenge is attractive as no change to, or treatment of, the crystals used is required to achieve the change, and the approach is universally applicable as it exploits the differing energy dependence of how X-rays interact with matter via elastic scattering, inelastic scattering or the photoelectric effect. The resulting benefits of higher energies are twofold. Firstly, as the X-ray energy increases the number of elastically scattered photons per unit absorbed dose, or diffraction efficiency (DE), increases, which is reflected experimentally in higher diffraction intensities for a given dose, implying an improved intensity-to-dose (*I*/*D*) ratio (Arndt, 1984 ▸; Fourme *et al.*, 2012 ▸; Helliwell *et al.*, 1993 ▸), meaning that crystal exposures can be reduced. Secondly, at higher energies photoelectron escape means that the energy deposited by X-rays can leave the crystal, depending on its volume, further reducing the absorbed dose (Nave & Hill, 2005 ▸). A key result from many years of work on radiation damage to cryocooled crystals is that X-ray-induced damage is proportional to the absorbed dose (Holton, 2009 ▸). Both of the effects introduced above predict higher diffracted intensities per unit absorbed dose at higher energies; thus, the dose can be reduced to obtain the same diffraction intensities. Consequently, the use of higher X-ray energies implies that more useful diffraction data can be collected from each crystal.

The energy dependence of DE was first noted by Arndt (1984 ▸), who showed that for crystalline proteins the probability of photoelectric absorption decreases more rapidly with increasing photon energy than does the probability of elastic scattering. The intensity of Bragg spots can be predicted using Darwin’s equation (Darwin, 1922 ▸), which takes X-ray beam and crystal parameters into account. A closer inspection of this also reveals a resolution dependence (Holton & Frankel, 2010 ▸), with gains in DE at higher X-ray energies being enhanced for higher resolution reflections.

In addition to the decrease in photoelectric absorption at high energies, another effect decreasing the deposited dose at higher energies is photoelectron escape. Nave & Hill (2005 ▸) simulated the tracks of photoelectrons in microcrystals and concluded that a significant proportion of photoelectrons could leave the crystal before causing damage (Nave & Hill, 2005 ▸). The inclusion of photoelectron escape and Compton scattering into calculation of the diffraction efficiency shows a theoretical fivefold gain in DE for 5 µm crystals when the energy of incident X-rays is increased from 7 to 30 keV (Cowan & Nave, 2008 ▸).

To date, attempts to demonstrate the benefits of high-energy data collection from protein crystals have been hampered by an absence of suitable detectors. Most experiments performed at high energies have been performed with CCD detectors (Fourme *et al.*, 2012 ▸; Jakoncic *et al.*, 2006 ▸; Shimizu *et al.*, 2007 ▸) partially combined with X-ray image intensifiers (Schiltz *et al.*, 1997 ▸), image plates (Jakoncic *et al.*, 2006 ▸; Schiltz *et al.*, 1997 ▸; Fourme *et al.*, 2011 ▸; Gonzalez *et al.*, 1994 ▸) or even point detectors (Müller *et al.*, 2002 ▸). All studies performed with two-dimensional detectors report that the detective quantum efficiency is rather low and not well characterized for high energies. Currently, most detectors for recording diffraction data at synchrotrons utilize a hybrid photon-counting (HPC) approach. HPC detectors feature an electronic counter bonded to a sensor which is typically made of silicon (Förster *et al.*, 2019 ▸). As the atomic number of silicon is low, the sensor rapidly becomes transparent as the X-ray energy is increased, with the result that the detector quantum efficiency (QE) falls as a function of energy: to less than 20% for a 450 µm silicon sensor at 25 keV (Donath *et al.*, 2013 ▸). Recently, detectors using cadmium telluride as a sensor material have been developed; the use of CdTe results in a detector QE of more than 90% below the cadmium absorption edge (26.7 keV) and of nearly 80% up to energies of 80 keV (Zambon *et al.*, 2018 ▸).

Simulations with *RADDOSE*-3*D* (Bury *et al.*, 2018 ▸), taking into account the quantum efficiency of a detector with a 750 µm thick CdTe sensor and assuming a top-hat beam profile, predict an optimal data-collection energy of 26 keV (Dickerson & Garman, 2019 ▸). Initial experiments with a PILATUS3 detector equipped with a CdTe sensor have recently been successfully used in MX to experimentally prove the benefits of photoelectron escape from microcrystals (Storm *et al.*, 2020 ▸), to collect data at 35 keV to ultrahigh resolution (Takaba *et al.*, 2019 ▸) and to investigate specific radiation damage at high energies (Ueno *et al.*, 2019 ▸).

Here, we present the first use of a CdTe EIGER2 detector for routine high-energy MX. The detector provides a nearly constant quantum efficiency across the investigated energy range. We experimentally demonstrate increased dose efficiency at higher energies, observing a more than twofold increase between 12.4 and 25 keV. No gain is observed when an identical experimental approach is employed using a silicon detector. We further observe an increase in the resolution of data obtained for a given absorbed dose at higher energies. The combination of these effects will allow fewer crystals to be used for structure determination and our results point to a high-energy future for synchrotron-based MX.

## 2. Methods   

### 2.1. Sample preparation and sample mounting   

Thermolysin crystals were grown in CrystalQuickX 96-well sitting-drop plates (Greiner) using a Mosquito crystallization robot (STP Labtech) at 20°C. 50 mg ml^−1^ lyophilized thermo­lysin (Sigma) was dissolved in 0.05 *M* MES pH 6, 45% DMSO, 50 m*M* NaCl and equilibrated against 1.2 *M* ammonium sulfate in a 1:1 ratio, with a final drop size of 200 nl. 50% ethylene glycol or 3 *M* ammonium sulfate was used as a cryoprotectant. The crystals were mounted in loops and then cryocooled. The dimensions of the rod-shaped crystals were measured using the data acquisition GUI (*GDA*) and ranged from 20 to 40 µm in diameter with lengths of 120–310 µm.

### 2.2. Beamline setup   

All experiments were carried out on beamline I24 at Diamond Light Source. A new cryogenic permanent-magnet undulator (CPMU) was installed shortly before the first experiments described here. Commissioning resulted in a varying X-ray flux between each experimental session. The X-ray flux was measured using a PD300-500 silicon PIN diode (Canberra), built by the Diamond Light Source detector group and calibrated by the Physikalisch-Technische Bundesanstalt (PTB) up to energies of 60 keV, at the start of each data-collection session. The diode response calibration provided by the PTB has an uncertainty of less than 1.3% at 12.5 keV and this decreases at higher energies to 1.0% at 25 keV. I24 features a two-stage focusing design with two pairs of Kirkpatrick–Baez mirrors; the first pair of mirrors features stripes with different coatings. For data collections below 20 keV a rhodium stripe was used and for experiments above 20 keV a platinum stripe was used. As the shape of the mirrors was optimized at 12.4 keV, the beam size increased when the platinum stripe was used. The secondary pair of mirrors were not translated during the experiments as the rhodium and platinum stripes overlap. Beam sizes at the sample position were determined by performing a knife-edge scan on a 200 µm thick gold wire and the full-widths at half-maximum (FWHMs) are given in Supplementary Table S2. Care was taken to measure beamline variables such as beam size and the photon flux prior to each data-collection session.

### 2.3. Detector setup   

Data were collected using an EIGER2 X 9M detector with a 750 µm thick CdTe sensor and a PILATUS3 X 6M detector with a 450 µm thick silicon sensor. The maximum frame rate of the PILATUS detector is 100 Hz (10 ms exposure times), while the EIGER detector can run at 230 Hz (4.3 ms). The detectors are mounted one above the other on a vertical translation stage, allowing data to be collected from each interchangeably. The sensor quantum efficiencies of each detector were interpolated from measured values provided by Dectris (Baden, Switzerland). To eliminate any concerns, and potential contributions to our results, from poor performance of cadmium telluride as a sensor material at low energies, comparative data were first collected using both detectors from selenium-soaked crystals above and below the selenium edge. These data, detailed in Supplementary Table S1 and Supplementary Fig. S1, show that the data from the CdTe EIGER at these energies are excellent, with differences arising from the smaller active area of the EIGER 9M compared with the PILATUS 6M. The data highlight the suitability of CdTe as a sensor material at conventional energies (*i.e.* around 12.4 keV) in addition to the primary use case of high-energy data collection where previously inaccessible elemental edges can be exploited.

### 2.4. Data collection   

For each data series, diffraction data were collected at 12.4, 17.5, 22.3 and 25 keV at a single position on a crystal. The order in which each energy was collected was varied between each data series and no more than one data series was collected at each position on a crystal. Doses were low and ranged between 300 and 800 kGy per data set. At each position on a crystal repeated wedges of 100° of diffraction data were collected, with a single wedge at each energy. The crystal-to-detector distance was varied such that the inscribed circle on the detector face corresponded to the same resolution at all energies. When multiple data series were collected from a single crystal the crystal was translated by at least twice the beam FWHM between each series. Data-collection parameters and measured fluxes are summarized in Supplementary Table S2.

To enable a direct comparison of the performance of the CdTe EIGER detector with that of the Si PILATUS detector, the exposure time was set to 10 ms in session A for data collected at 22.3 keV or below. In subsequent sessions, where the PILATUS was not used for comparison, an exposure time of 5 ms was used. In session A, at least one set of collections at the four energies was performed with both detectors at different positions on the same crystal. For the data at 25 keV, exposure times up to 83 ms at full transmission were required to compensate for the lower flux as well as the lower scattering efficiency. While this approach results in a slower angular velocity at 25 keV, we do not believe that this impacts the observed differences in data quality or the intensity per unit absorbed dose. To be able to calculate the flux as accurately as possible, intensity values from an X-ray beam-position monitor close to the sample position were recorded for all data sets. In session D, higher energies became accessible due to the ongoing commissioning of the CPMU, although exposures of 0.7 s per data frame were required at 27 keV. As commissioning continues it is anticipated that higher fluxes, and hence reduced exposure times, will become accessible, with any time penalty associated with high-energy data collection removed. To allow the energy dependence of the diffracted intensity per unit absorbed dose, 〈*I*〉/*D*, at high resolution to be probed, exposure times were increased and a shorter crystal-to-detector distance was used during this session.

### 2.5. Data processing   

Data were processed with the *DIALS* integration package (version 3.0.4; Winter *et al.*, 2018 ▸). To generate statistics with consistent parameters across data sets, *xia*2 (Winter, 2010 ▸) was used. Raw intensities were used from the data-processing integration step to avoid complications introduced by scaling routines or inadvertent ‘scaling out’ of energy-dependent differences.

Crystals typically diffracted to 1.4–2.0 Å resolution, taking into account diffraction in the corners of the detector and applying the CC_1/2_ > 0.33 criterion. However, a resolution cutoff of 2.0 Å was applied to all data when quantifying energy-dependent changes in the mean diffracting power. This was defined by the maximum resolution of the inscribed circle at the minimum detector distance at 12.4 keV.

The average diffraction-weighted dose, referred to here as dose, was calculated for each data set using *RADDOSE*-3*D* (Bury *et al.*, 2018 ▸). Dose calculations assumed that the long axis of the crystal was perpendicular to the X-ray beam and parallel to the rotation axis of the I24 vertical goniometer. The 〈*I*〉/*D* value is defined as the unscaled mean intensity given by *xia*2 from the lowest resolution shell up to 2.0 Å divided by the deposited dose estimated for the individual data set.

## 3. Results and discussion   

In order to quantify the energy dependence of the diffraction efficiency of protein crystals, 29 low-dose data series were collected from 11 thermolysin crystals. Each data series consisted of four 100° wedges collected from the same position on a crystal, with each wedge recorded using a different X-ray energy (12.4, 17.5, 22.3 or 25 keV). 22 data series were recorded using an EIGER2 9M detector (750 µm thick CdTe sensor) and seven with a PILATUS3 6M detector (450 µm thick Si sensor). In order to minimize the contribution of radiation damage to the observed trends, the total absorbed dose was kept low: to <800 Gy per sweep (and <2 MGy per entire data series), *i.e.* less than 5% of the 43 MGy required to halve the diffracting power of cryocooled crystals (Owen *et al.*, 2006 ▸). The order of energies was varied between each series and no more than one data series was collected from each position on a crystal.

**Figure 1:**
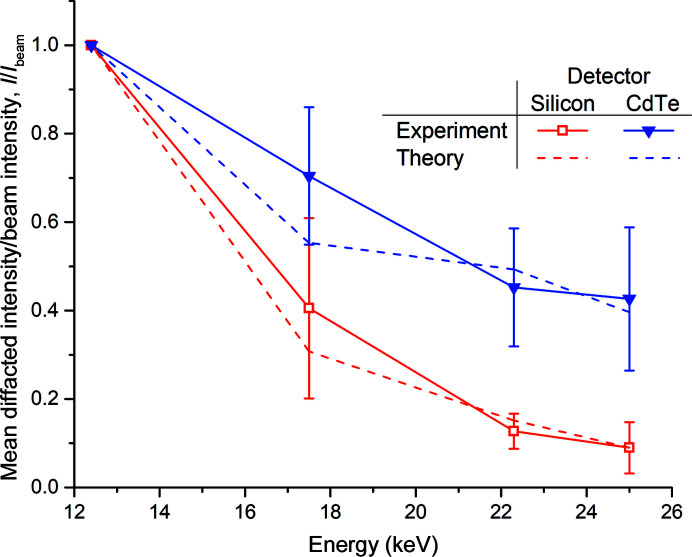
Ratio of diffracted intensity to beam intensity (*I*/*I*_beam_) as a function of energy for data recorded using both the CdTe EIGER and Si PILATUS detectors. Experimental data are the mean of 22 and seven data sets for the EIGER and PILATUS detectors, respectively. The predicted energy dependence of *I*/*I*_beam_ determined from theory using the measured fluxes and beam sizes is overlaid. To highlight the energy dependence of *I*/*I*_beam_, the data are normalized to 12.4 keV.

Fig. 1 ▸ shows the agreement between the observed diffracted intensity divided by the beam intensity (*I*/*I*_beam_) as a function of energy for the crystals used in this study and *I*/*I*_beam_ predicted by Darwin’s equation, which is presented in a simple form by Giacovazzo *et al.* (2011 ▸)

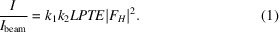

Here, *k*_1_ = *e* ^4^/*m* ^2^ *c* ^4^ takes into account universal constants, and 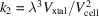 , where λ is the X-ray wavelength, *V*_xtal_ is the illuminated crystal volume and *V*_cell_ is the volume of the unit cell. *L* is the Lorentz factor, *P* is the polarization factor, *F_H_* is the structure factor, and *T* and *E* are the X-ray transmission and extinction coefficients of the crystal, respectively. The analytical expression for the product of 〈*LP*〉*f*_obs_ = π(3 + cos4θ)/16sinθ derived by Holton & Frankel (2010 ▸) can be used, where *f*_obs_ represents the fraction of reciprocal-lattice points accessible while the crystal rotates, and energy-independent terms excluded to show

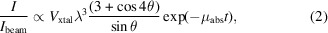

where θ is the Bragg angle, *t* is the crystal thickness and μ_abs_ is its X-ray absorption coefficient. When applying this expression to the data collected here, we also consider the energy-dependent quantum efficiency of the detector used.

The agreement between experiment and theory shown in Fig. 1 ▸ illustrates that the ratio of elastically scattered photons to incident photons varies as expected for both detectors and, importantly, gives confidence in both the accuracy and validity of the quantity mean diffracted intensity as the numerator *I* in the ratio 〈*I*〉/*D* derived below. We note that the λ^2^ variation in *I*/*I*_beam_ that might be expected from (2) (as the 〈*LP*〉*f*_obs_ term is almost directly proportional to the wavelength) is not observed in Fig. 1 ▸ due to the variation in the beam size (and hence *V*_xtal_) between energies, as discussed in Section 2.2.

**Figure 2:**
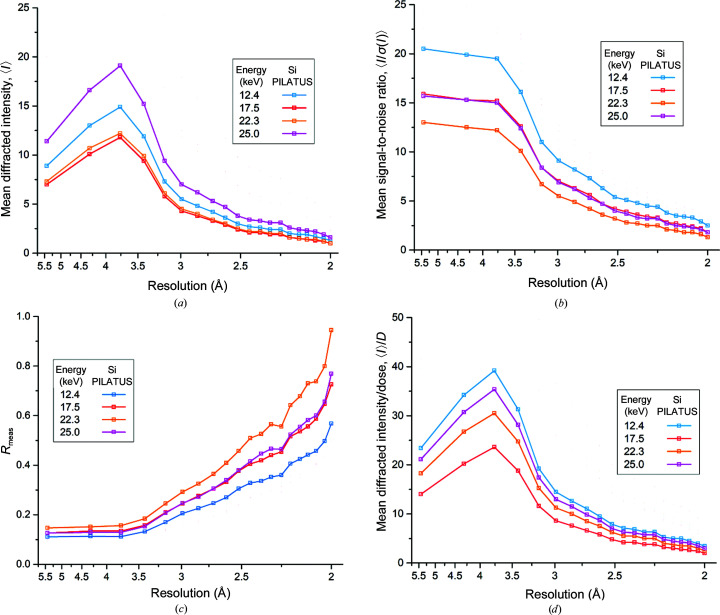
Overview of a data series collected using the Si PILATUS detector, illustrating the impact of higher X-ray energies and detector sensor material. (*a*) shows the mean unscaled intensity per Bragg spot 〈*I*〉 as a function of resolution. (*b*) Mean unscaled signal-to-noise ratio per Bragg spot 〈*I*/σ(*I*)〉. (*c*) Multiplicity-independent merging *R* value *R*_meas_. (*d*) Observed mean diffracted intensity per Bragg spot normalized to the absorbed dose, 〈*I*〉/*D*. In all panels the data points reflect the high-resolution limit of each shell; the lowest resolution data point includes reflections over the range 40–5.5 Å.

**Figure 3:**
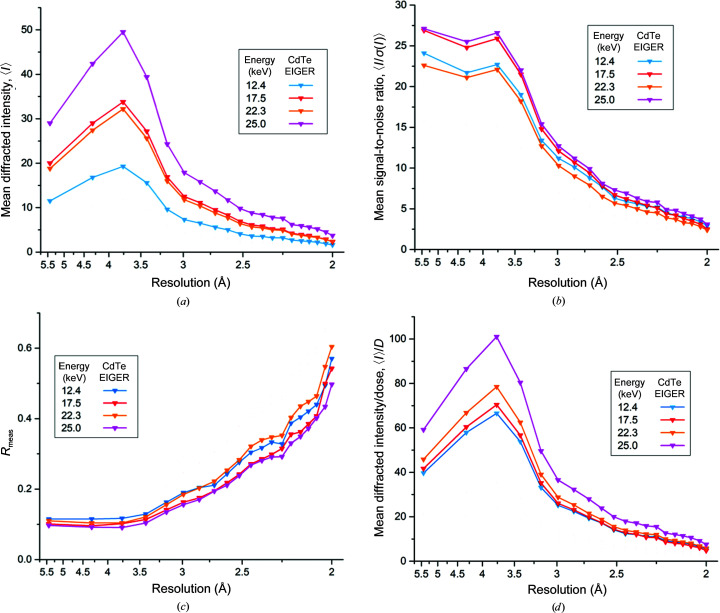
Overview of a data series collected using the CdTe EIGER detector, illustrating the impact of higher X-ray energies and detector sensor material. (*a)* shows the mean unscaled intensity per Bragg spot 〈*I*〉 as a function of resolution. (*b*) Mean unscaled signal-to-noise ratio per Bragg spot 〈*I*/σ(*I*)〉. (*c*) Multiplicity-independent merging *R* value *R*_meas_. (*d*) Observed mean diffracted intensity per Bragg spot normalized to the absorbed dose, 〈*I*〉/*D*. In all panels the data points reflect the high-resolution limit of each shell; the lowest resolution data point includes reflections over the range 40–5.5 Å.

Intensity statistics for two individual data series collected from the same crystal, one recorded using each detector, are shown in Fig. 2 ▸ (Si PILATUS data) and Fig. 3 ▸ (CdTe EIGER data). The same figure with PILATUS and EIGER data overlaid for direct comparison is presented in Supplementary Fig. S2. Both of these data series were collected with the same data-collection parameters and the same increasing energy sequence (12.4, 17.5, 22.3 and finally 25 keV). The mean unscaled intensities recorded over the two data series while aiming at keeping the total diffracted intensity approximately constant are shown in Figs. 2 ▸(*a*) and 3 ▸(*a*). A strong energy dependence is clear for data recorded using the CdTe EIGER detector, with higher intensities recorded at higher energies, a trend that was not observed using the Si PILATUS detector. The observed signal-to-noise ratio, 〈*I*/σ(*I*)〉, is shown in Figs. 2 ▸(*b*) and 3 ▸(*b*), and this again illustrates the advantage conferred by cadmium telluride. The CdTe data again show a constant increase as a function of energy, whereas the maximum for the Si data is at 17.5 keV: at 22.3 and 25 keV, when the absorption of silicon is low, 〈*I*/σ(*I*)〉 falls. Concomitant with the decrease in the quantum efficiency of the silicon sensor, the internal consistency of the data also decreases, as can be seen from the increased *R*_meas_ values for data collected with the Si PILATUS detector at higher energies [Figs. 2 ▸(*c*) and 3 ▸(*c*)]. We note that other detector properties beyond the sensor material contribute to some of the observed differences: both the smaller pixel size and the zero deadtime of the EIGER detector also affect data quality (Casanas *et al.*, 2016 ▸). The 0.95 ms dead time of the PILATUS detector used corresponds to almost 10% of the total exposure time for data collected at energies below 25 keV, for example. This deadtime and the larger QE of a 750 µm CdTe sensor compared with a 450 µm Si sensor combine to result in the higher spot intensities observed using the EIGER at 12.4 keV in Supplementary Fig. S2 (the quantum efficiencies of both detector sensors used in this work are given in Supplementary Tables S2 and S3). Our results clearly show that a high-*Z* sensor material such as CdTe is essential to exploit the benefits of high-energy data collection and offers few, if any, disadvantages at more traditional energies around 12.4 keV/1 Å.

With doses of less than 540 kGy per data set (Supplementary Tables S2 and S3), global radiation damage is unlikely to have a significant effect on the observed trends. Indeed, 2*F*_o_ − *F*_c_ maps comparing data sets collected at the same position in the crystal display almost no signs of site-specific radiation damage (Supplementary Fig. S3).

**Figure 4:**
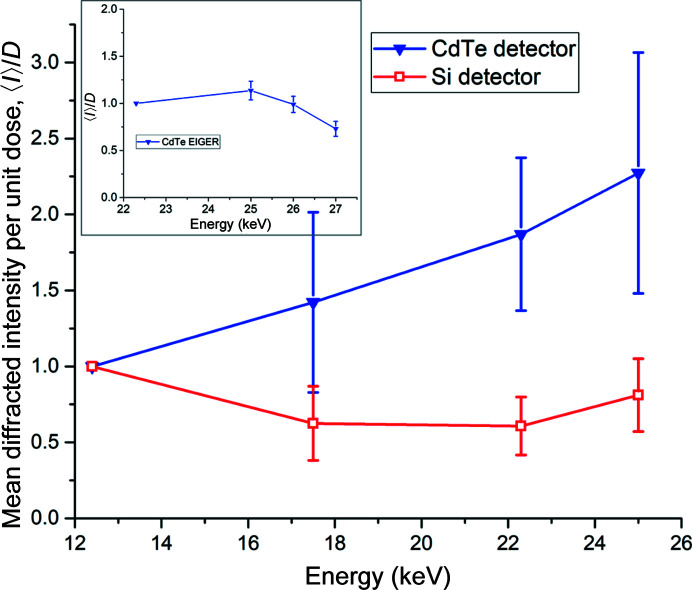
Increase in the diffracted intensity per unit absorbed dose, 〈*I*〉/*D*, as a function of energy. Data shown are averaged from 29 data series (22 recorded using the CdTe EIGER and seven using the Si PILATUS), with the standard deviation at each energy shown as error bars. The variation in 〈*I*〉/*D* over an energy range of 22.3–27 keV observed in an additional five data series is shown in the inset. The data shown in the inset are normalized to 22.3 keV. The mean diffracted intensity, 〈*I*〉, is scaled to the illuminated crystal volume defined by the measured beam sizes given in Supplementary Table S2.

Figs. 2 ▸(*d*) and 3 ▸(*d*) show the diffracted intensity per unit absorbed dose, 〈*I*〉/*D*, for the two data series. When the CdTe EIGER detector, optimized for high-energy data collection, is used a clear increase in 〈*I*〉/*D* is observed at 22.3 and 25 keV. Some of this gain is due to the change in beam size and hence illuminated crystal volume at 22.3 and 25 keV (Section 2.2 and Supplementary Table S2). Fig. 4 ▸ shows 〈*I*〉/*D* per crystal volume as a function of energy, scaling the numerator by the illuminated volume using beam sizes measured prior to data collection. The trend of increasing diffracted intensity per unit absorbed dose is still clearly observed for all crystals: over 22 data series, the mean increase in 〈*I*〉/*D* between 12.4 and 25 keV is a factor of 2.3 (Fig. 4 ▸). When using a detector with a silicon sensor no such increase is observed, with 〈*I*〉/*D* being approximately constant over the energy range used and an energy of 12.4 keV proving optimal. Five additional data series were collected from two crystals over a higher X-ray energy range (22.3–27 keV), as the optimal energy for data collection using a CdTe-based detector is expected to be at around 26 keV (Dickerson & Garman, 2019 ▸). Over this limited energy range we observe no clear peak in the diffracted intensity per unit dose, with 〈*I*〉/*D* being approximately constant between 22 and 26 keV, although a significant decrease, beyond experimental error, is observed at 27 keV as expected (Fig. 4 ▸, inset).

While an increase in diffraction efficiency as a function of energy is predicted by theory (Arndt, 1984 ▸), the size of the gain observed in these experiments is larger than that predicted by Dickerson & Garman (2019 ▸) by ∼40%. There are other experimental factors which act to increase the gains realized for high-energy data collection. Firstly, the beam used in these experiments has a Gaussian rather than a top-hat profile. The theoretical increase of 1.6 in DE between 12.4 and 25 keV predicted by Dickerson and Garman assumes a top-hat beam, with gains of 3 to 4 only realized for beam and crystal sizes of less than 2 µm. When a Gaussian beam is used, photoelectrons will not be generated evenly within the illuminated volume, which is partly reflected in an increased theoretical diffraction efficiency of 1.9 between 12.4 and 25 keV when this is factored into the dose calculation. Some additional gain may therefore result as photoelectrons migrate out of the central high-dose region, aided by the longer path lengths of photoelectrons at higher energies. Secondly, one has to consider that despite best efforts, small errors in the measurement of the X-ray beam size and intensity and crystal size can easily result in underestimation or overestimation of the absorbed dose and hence *I*/*D*. We sought to minimize this as a source of error through the use of multiple crystals and data collection over multiple sessions with the flux and beam size measured at each.

Some studies postulate that intensities at higher energies can be enhanced relative to lower energies due to a reduced background per unit area by the inverse-square law and lower sample absorption (Fourme *et al.*, 2012 ▸; Helliwell *et al.*, 1993 ▸). Here, we choose to keep the resolution at the detector edge constant, moving the detector further away from the crystal at higher energies, since this is how crystallographers will best exploit the energy-dependent gains in real-world data collection. An increase in the mean spot size is observed with increasing energy (and sample-to-detector distance), consistent with the I24 beam divergence, while background intensity also increases. The latter, whilst on the face of it counterintuitive, increases at a lower rate than the spot intensities and is consistent with elastic scattering from the noncrystalline component of the sample becoming the main factor, since it is concentrated at smaller angles with increasing beam energy (Gonzalez *et al.*, 1994 ▸). Our observations suggest that variation in spot size and background intensity are not significant influences on the improvements in the metrics of diffraction data quality seen at higher energies.

In the above, photoelectron escape has not been considered when calculating absorbed doses. While this effect is negligible at 12.4 keV, at higher energies photoelectrons can escape from the illuminated volume, reducing the effective deposited dose. At 25 keV the deposited dose is reduced by 20% for 20 µm crystals to *D*_PE_ (*D* and *D*_PE_ are calculated using *RADDOSE*-3*D* and are shown in Supplementary Table S3). Compared with the twofold increase in 〈*I*〉/*D* shown in Fig. 4 ▸, 〈*I*〉/*D*_PE_ shows a further increase as a function of energy, increasing by almost a factor of 3 between 12.4 and 25 keV (Supplementary Fig. S4). However, due to the crystal size, photoelectron escape is assumed to be a minor effect here. Future experiments to quantify photoelectric escape and validate the optimal energy of 26 keV predicted by Dickerson and Garman should focus on much smaller crystals.

**Figure 5:**
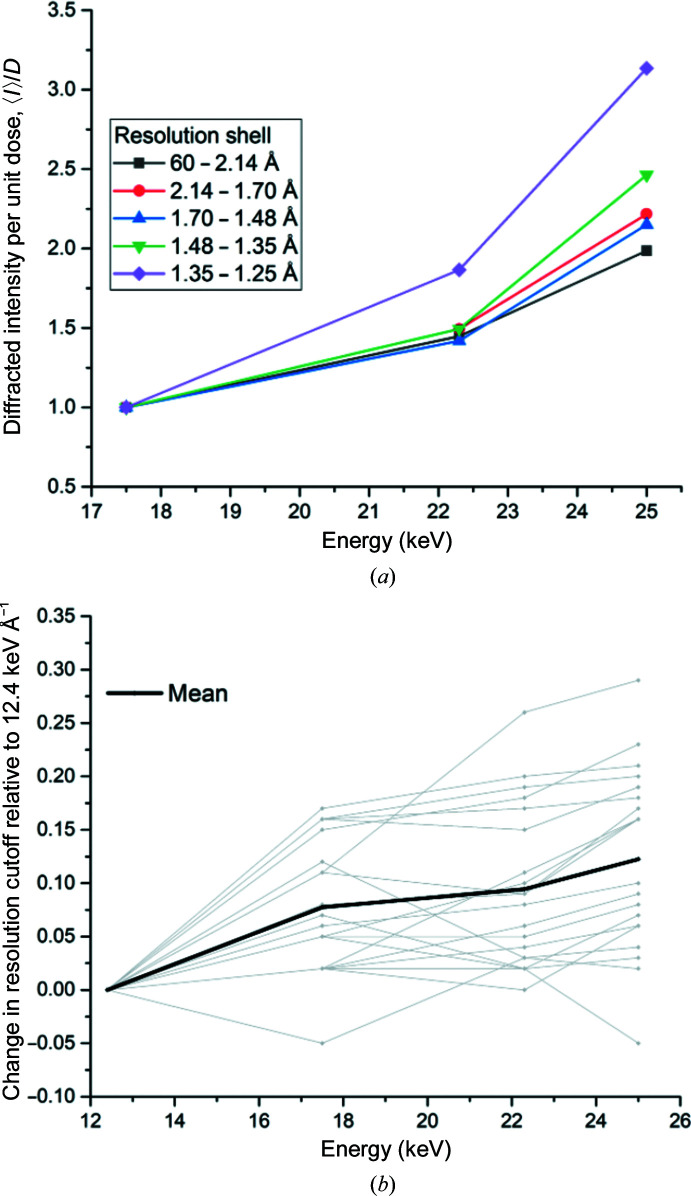
(*a*) Resolution dependence of the diffracted intensity per unit absorbed dose, 〈*I*〉/*D*. To allow direct comparison of different resolution shells, 〈*I*〉/*D* in each resolution shell is normalized to the value at 17.5 keV. The mean diffracted intensity, 〈*I*〉, is scaled to the illuminated crystal volume. (*b*) Energy-dependent change in resolution cutoff as ascertained using a criterion of CC_1/2_ > 0.33 for 20 crystals. The mean change in resolution is shown as a thick black line.

To experimentally confirm the predicted resolution dependence of the gains in diffracted intensity per unit dose, high-resolution (1.25 Å) data series were also collected. The dimensions of the EIGER X 9M and the currently achievable minimum crystal-to-detector distance at I24 preclude data collection to this resolution at 12.4 keV, so these data series were normalized to 17.5 keV. Fig. 5 ▸(*a*) shows the mean diffracting power per unit absorbed dose, 〈*I*〉/*D*, in different resolution shells as a function of energy for data from one crystal recorded using the CdTe EIGER. A clear resolution dependence is observed, with 〈*I*〉/*D* increasing by a factor of 2.0 for the lowest resolution shell compared with 3.1-fold over the range 1.35–1.25 Å. It follows that the high-resolution limit of the diffraction data should also increase at higher energies.

Fig. 5 ▸(*b*) shows the energy-dependent resolution cutoff for 20 data series with diffraction to between 2.1 and 1.65 Å resolution at 12.4 keV. For two data series the resolution cutoff could not be determined for all energies automatically and so they were not included in this comparison. The resolution limit was determined using *dials.scale*, applying a cutoff criterion of CC_1/2_ > 0.33. A steady increase in resolution was observed as a function of energy, with gains of 0.08, 0.09 and 0.12 Å at 17.5, 22.3 and 25 keV, respectively. By running a pairwise *t*-test based on these data, the low *p*-values for these changes in resolution (8.4 × 10^−6^, 6.2 × 10^−6^ and 1.3 × 10^−6^) illustrate that the gains, although modest in size, are statistically significant. Resolution gains can also clearly be observed in the electron-density maps obtained (Supplementary Fig. S5). Reduced air scatter and lower absorption at high X-ray energies also act to explain the varying gain in resolution. A more systematic investigation of this phenomenon including crystals from different proteins is required. With the advancement of synchrotron technology, from undulators providing high photon fluxes at high energies through to large area detectors that are able to rapidly record high-energy photons, it is now possible to efficiently and routinely collect data at energies which are optimal for macromolecular crystallography. Within this study, we show experimentally that a CdTe-based detector enables the increased diffraction efficiency of crystals at high X-ray energies to be exploited due to its increased quantum efficiency in comparison to silicon-based detectors. The energy dependence of intensities follows Darwin’s law in both cases. The benefits of data collection at 25 keV include improved data statistics that can be recorded for a given absorbed dose and also include an increase in the resolution of data compared with data collected at 12.4 keV. This increase in information content can, for example, enable the identification of water molecules in structural enzymology and make a critical difference in understanding the structural function of proteins.

These results impact on all macromolecular crystallography experiments from rotation to serial even when the crystal sizes used are relatively modest (∼20 µm) and should be considered in the design of future MX beamlines and for X-ray data collection from all samples that yield crystals of limited size.

## 4. Related literature   

The following reference is cited in the supporting information for this article: Liebschner *et al.* (2019 ▸).

## Supplementary Material

Supplementary Tables and Figures. DOI: 10.1107/S2052252521008423/jt5058sup1.pdf
