# Supplementary material for: Experimental evidence for the benefits of higher X-ray energies for macromolecular crystallography
Source: IUCrJ. 2021 Sep 9;8(Pt 6):896–904. doi: 10.1107/S2052252521008423 (PMC8562668; doi:10.1107/S2052252521008423)
Supplement: Supplementary file 1 [file m-08-00896-sup1.pdf]

IUCrJ

Volume 8 (2021)

Supporting information for article:

Experimental evidence for the benefits of higher X-ray energies for macromolecular crystallography

Selina L. S. Storm, Danny Axford and Robin L Owen

Table S1 Comparative data from a selenium-soaked thaumatin crystal (space group $P4_12_12$) collected using the CdTe Eiger 9M and Si Pilatus 6M

Data were collected with the X-ray beam defocused to 50×50 μm^2, 10 ms images, 0.1 deg oscillation per image, 360 deg total oscillation. 1% transmission and the detector at 200 mm.

Detector	CdTe Eiger	CdTe Eiger	Si Pilatus	Si Pilatus
Energy / keV	12.40	12.673	12.40	12.673
Wavelength (Å)	1.000	0.9784	1.000	0.9784
Resolution range (Å)	53.81 - 1.44, 53.85-3.91, 1.47 - 1.44	57.72 -1.41, 57.72-3.83, 1.44 -1.41	149.77-1.24, 150.87-3.37, 1.26-1.24	149.75-1.33, 150.71-3.61, 1.35-1.33
Observations	725163, 59610, 428	776077, 63712, 533	1355644, 92858, 11820	1291849, 3284, 2797
Completeness (%)	86.14, 100, 13.86	86.61, 100, 16.52	97.17, 100, 76.01	99.83, 100, 96.61
Multiplicity	18.0, 22.7, 1.3	18.1, 22.9, 1.3	19.3, 23.2, 4.4	21.9, 22.9, 9.6
CC-half	0.997, 0.993, 0.444	0.997, 0.996, 0.206	0.999, 0.999, 0.337	0.998, 0.999, 0.321
I/sigma	18.7, 56.7, 0.9	14.3, 51.4, 0.3	11.3, 55.6, 0.5	10.2, 51.9, 0.4
R_{meas}	0.107, 0.055, 0.810	0.133, 0.067, 1.777	0.158, 0.060, 1.649	0.207, 0.068, 2.711
R_{pim}	0.022, 0.011, 0.529	0.028, 0.014, 0.206	0.033, 0.012, 0.748	0.043, 0.014, 0.865

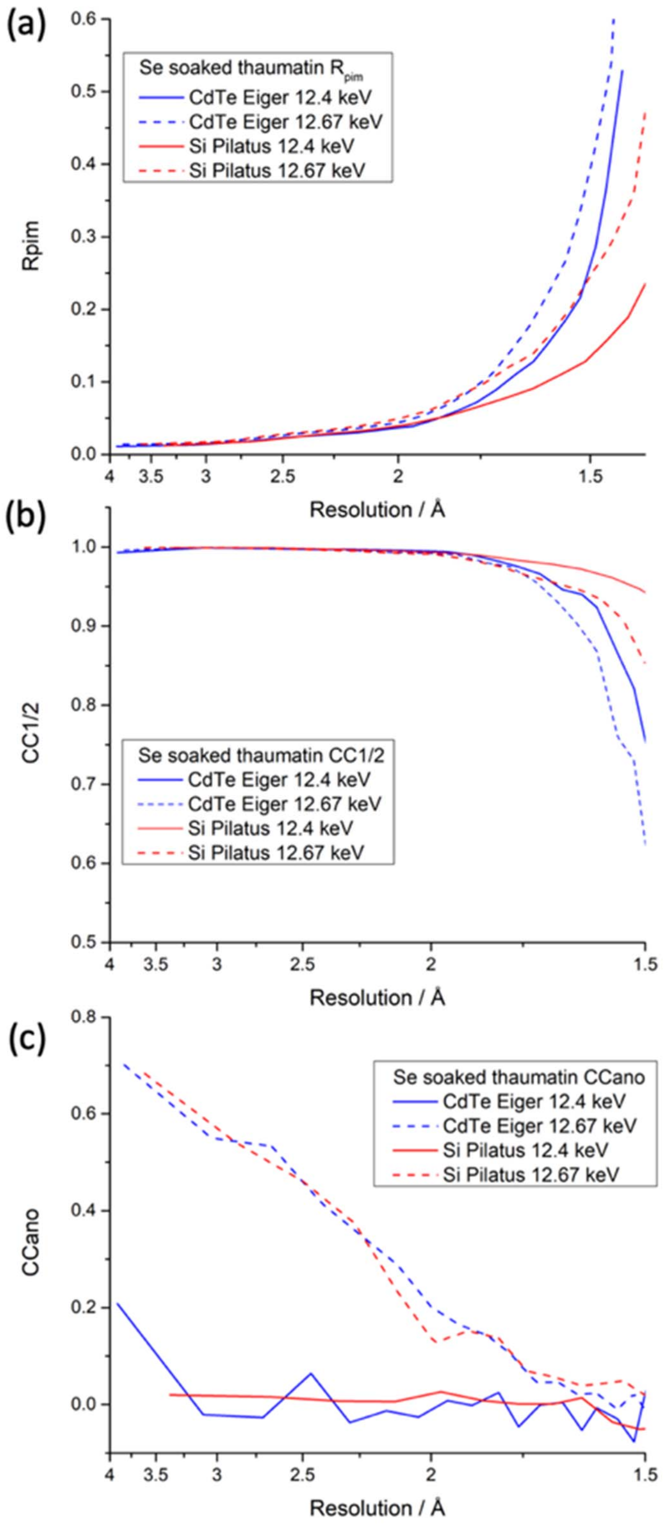

Figure S1 Plots of scaling statistics for the data summarised in table S1. (a) R_{pim}, (b) $CC_{1/2}$ and (c) CC_{ano}.

Table S2 Overview of all diffraction data collected

A data series refers to a set of four datasets collected at four different energies from a single position on a crystal. No more than one data series was collected at each position on a crystal. Doses were calculated using *RADDOSE-3D* (Bury *et al*., (2020)) taking photoelectron escape into account.

Session & number of data series	Energy / keV	Incident flux / $\times 10^{11}$ ph s^{-1}	Beamsize / μm^2	Crystal diameter / µm	Total exposure time per dataset / s	Total deposited dose per dataset / kGy
**A** 8 CdTe 7 Si	12.4	26.40	7.9 × 8.9	25	10	280
	17.5	8.48		25	10	440
	22.3	4.09	9.7 × 10.2	25	10	350
	25.0	0.255		25	83	400
**B** 8 CdTe	12.4	52.80	7.0 × 8.1	25	5	390
	17.5	17.00		25	5	590
	22.3	4.80	9.0 × 10.6	25	5	510
	25.0	0.32		25	70	500
**C** 6 CdTe	12.4	46.8	7.9 × 9.1	20	5	400
	17.5	18.0		20	5	570
	22.3	4.52	9.0 × 10.7	20	5	590
	25.0	0.32		20	70	530
**D** 5 CdTe	22.3	5.06	9.0 × 10.7	25	5	580
	25	0.31		25	70	510
	26	0.26		25	90	470
	27	0.04		25	700	500
**E** 5 CdTe	17.5	18.0	7.9 × 9.1	30	8	840
	22.3	4.52	9.0 × 10.7	30	8	840
	25	0.32		30	110	700

Table S3 Si Pilatus data collection parameters and integration statistics obtained for the two data series shown in figures 2, 3 and S2

Note that <I> and <I/σ(I)> reported are for unscaled data allowing direct comparison between independent datasets. $CC_{1/2}$ and R_{meas} reported are for scaled data as conventionally reported as indicators of dataset quality. All statistics are given in the form (overall, low resolution, high resolution). Statistics given are for over the entire resolution range with values in brackets are for the outermost resolution shell (2.0-2.03 Å). The resolution cut was set to 2 Å, as the inscribed circle at 12.4 keV is limited to this resolution by the detector area. The resolution given in the table was calculated based on the $CC_{1/2}$ > 0.33 criterion and takes reflections in the corners of the detector into account. The dose refers to the diffraction weighted dose obtained using RADDOSE3D as discussed in the text. The data collection sequence for both crystals was 12.4 keV, 17.5 keV, 22.3 keV, 25 keV

Data series	2	2	2	2
Energy	12.4	17.5	22.3	25.0
Detector	Si Pilatus	Si Pilatus	Si Pilatus	Si Pilatus
Detector QE	0.835	0.475	0.264	0.193
Resolution range / Å	60-2.0, 60-5.4, 2.03-2.00			
N obs	326214, 16409, 15841	328963,16457, 16139	238551, 11683,12174	240791, 11715, 12298
<I>	5.0, 8.9, 1.3	3.9, 7.0, 1.0	4.1, 7.3, 1.0	6.3, 11.4, 1.6
<I/σ(I)>	8.3, 20.5, 2.5	6.3, 15.9, 1.8	5.0, 13.0, 1.3	6.2, 15.7, 1.8
R_{meas}	0.244, 0.114, 0.910	0.303, 0.131,1.167	0.316, 0.147,0.945	0.249, 0.119, 0.733
R_{pim}	0.074, 0.037, 0.275	0.092, 0.041, 0.350	0.096, 0.047, 0.282	0.079, 0.040,0.229
$CC_{1/2}$	0.992, 0.992, 0.814	0.989, 0.994, 0.710	0.986, 0.991, 0.791	0.990, 0.993, 0.861
Incident flux ph/s	2.88×10^{10}	7.88×10^{10}	1.35×10^{11}	2.76×10^{10}
total exposure / s	5	5	5	83
Dose / kGy	380	500	400	540
Deposited dose /kGy	370	460	350	430

Table S4 CdTe Eiger data collection parameters and integration statistics obtained for the two data series shown in figures 2, 3 and S2

Note that <I> and <I/σ(I)> reported are for unscaled data allowing direct comparison between independent datasets. $CC_{1/2}$ and R_{meas} reported are for scaled data as conventionally reported as indicators of dataset quality. All statistics are given in the form (overall, low resolution, high resolution). Statistics given are for over the entire resolution range with values in brackets are for the outermost resolution shell (2.0-2.03 Å). The resolution cut was set to 2 Å, as the inscribed circle at 12.4 keV is limited to this resolution by the detector area. The resolution given in the table was calculated based on the $CC_{1/2}$ > 0.33 criterion and takes reflections in the corners of the detector into account. The dose refers to the diffraction weighted dose obtained using RADDOSE3D as discussed in the text. The data collection sequence for both crystals was 12.4 keV, 17.5 keV, 22.3 keV, 25 keV.

Data series	1	1	1	1
Energy	12.4	17.5	22.3	25.0
Detector	CdTe Eiger	CdTe Eiger	CdTe Eiger	CdTe Eiger
Detector QE	0.912	0.933	0.940	0.945
Resolution range / Å	60-2.0, 60-5.4, 2.03-2.00			
N obs	239882, 12598, 12117	239717,12433, 12160	239887,12401, 12341	243415, 12520, 12379
<I>	6.5, 11.5, 1.6	11.1, 20.0, 2.3	10.5, 18.8, 2.4	16.2, 29.0, 3.7
<I/σ(I)>	9.7, 24.1, 2.9	10.5, 26.9, 2.5	8.9, 22.6, 2.4	11.0, 27.1, 3.1
R_{meas}	0.191, 0.115, 0.570	0.171, 0.101, 0.542	0.196, 0.110, 0.604	0.116, 0.068, 0.352
R_{pim}	0.135, 0.081, 0.403	0.121, 0.072, 0.383	0.139, 0.078, 0.427	0.116, 0.068, 0.352
$CC_{1/2}$	0.958, 0.965, 0.640	0.969, 0.978, 0.686	0.961, 0.976, 0.622	0.977, 0.979, 0.714
Incident flux ph/s	2.22×10^{10}	7.56×10^{10}	1.37×10^{11}	2.54×10^{10}
total exposure / s	5	5	5	83
Dose / kGy	290	480	410	490
Deposited dose /kGy	280	440	350	400

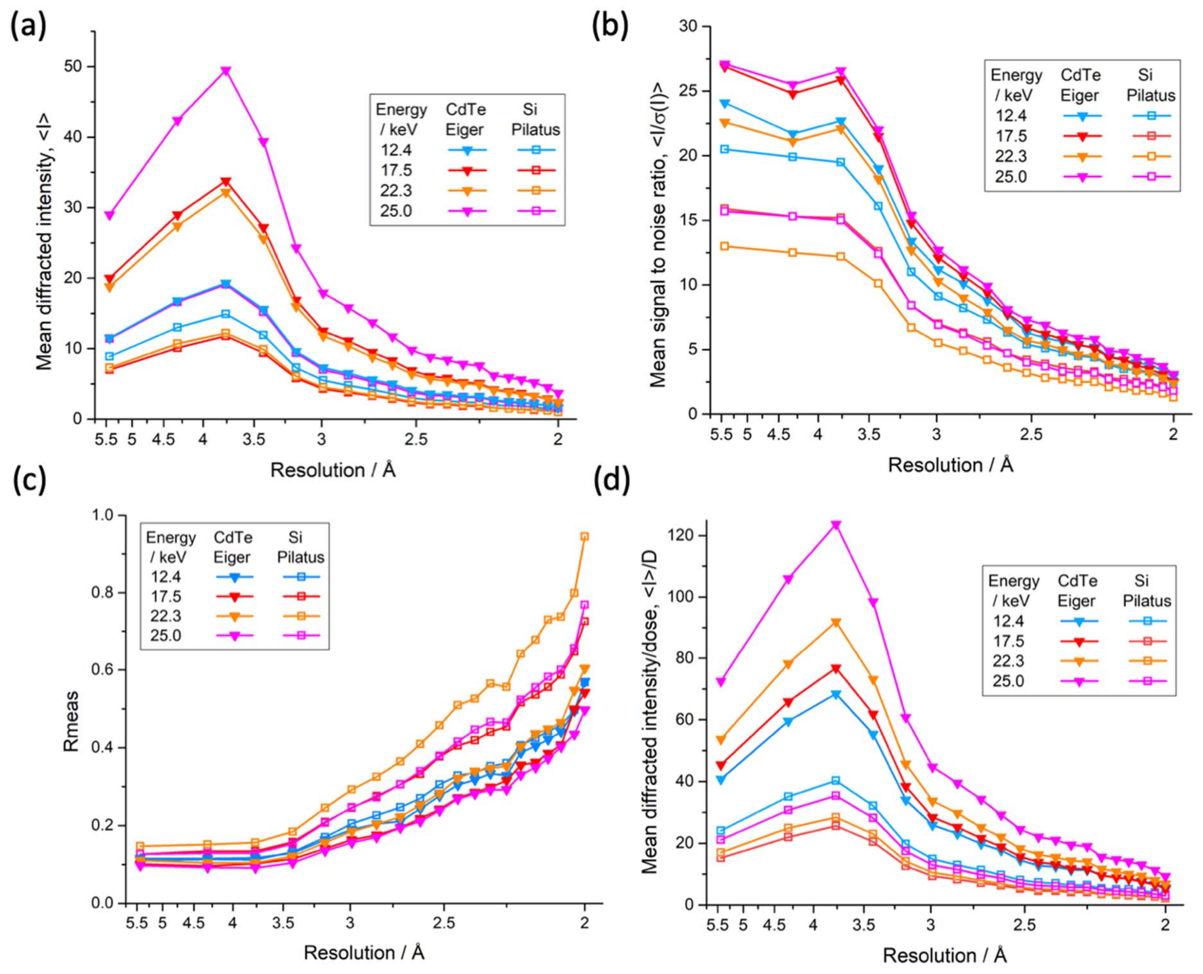

Figure S2 Overlay of data shown in figures 2 and 3 allowing direct comparison of data series collected using Si Pilatus and CdTe Eiger data series. Si Pilatus detector are shown as hollow squares while CdTe Eiger data are shown as filled triangles. (a) shows the mean unscaled intensity per Bragg spot <I>, as a function of resolution (b) Mean unscaled signal to noise ratio per Bragg spot <I/σ(I)>. (c) Redundancy independent merging R value R_{meas}. (d) Observed mean diffracted intensity per Bragg spot normalized to the absorbed dose, <I>/D. In all panels data points reflect the high resolution limit of each shell, the lowest resolution data point includes reflections over the range 40 – 5.5 Å.

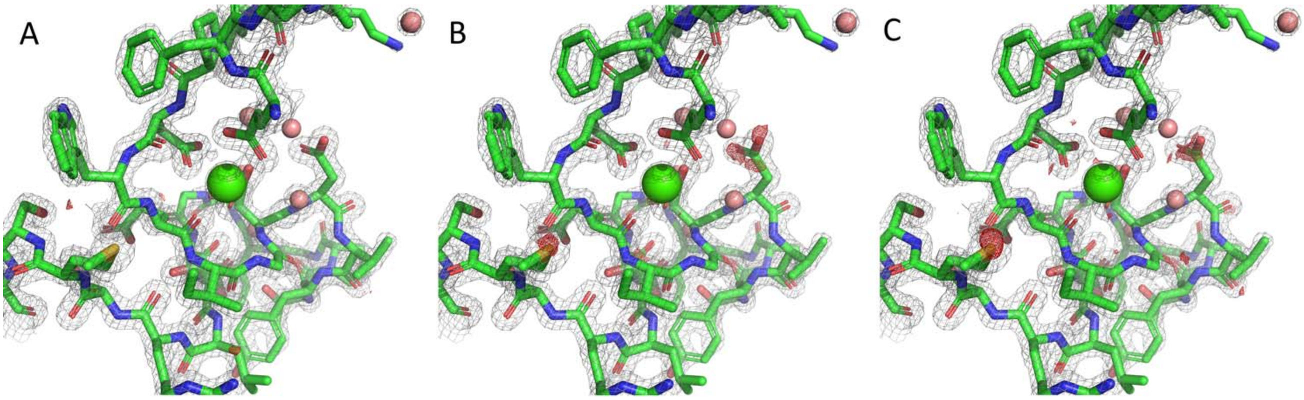

Figure S3 Electron density obtained from sequential datasets forming a data series showing worst case site-specific radiation damage. The grey mesh represents the 2Fo-Fc map and the stick model the refined thermolysin structure from the first dataset in the series (17.5 keV). These data were collected in the order: 17.5 keV, 22.3 keV, 25.0 keV, 12.4 eV. In A, B, and C Fo-Fo difference maps have been superimposed onto the refined data, shown in red/green dense mesh and contoured to 3.5 sigma. A shows 12.4 keV minus 25.0 keV, B shows 12.4 keV minus 22.3 keV, C shows 12.4 keV minus 17.5 keV. Maps produced with phenix.fobs_minus_fobs_map. Very slight density loss (red) is visible on acidic residue ASP191 and the sulphur atom of MET205 when differences are inspected across the sequence of 4 datasets. We note that these are the only notable site specific changes visible and the rest of the structure is very flat regards difference density Data were recorded using the CdTe Eiger detector and processed using phenix.refine (Liebschner *et al*. (2019)).

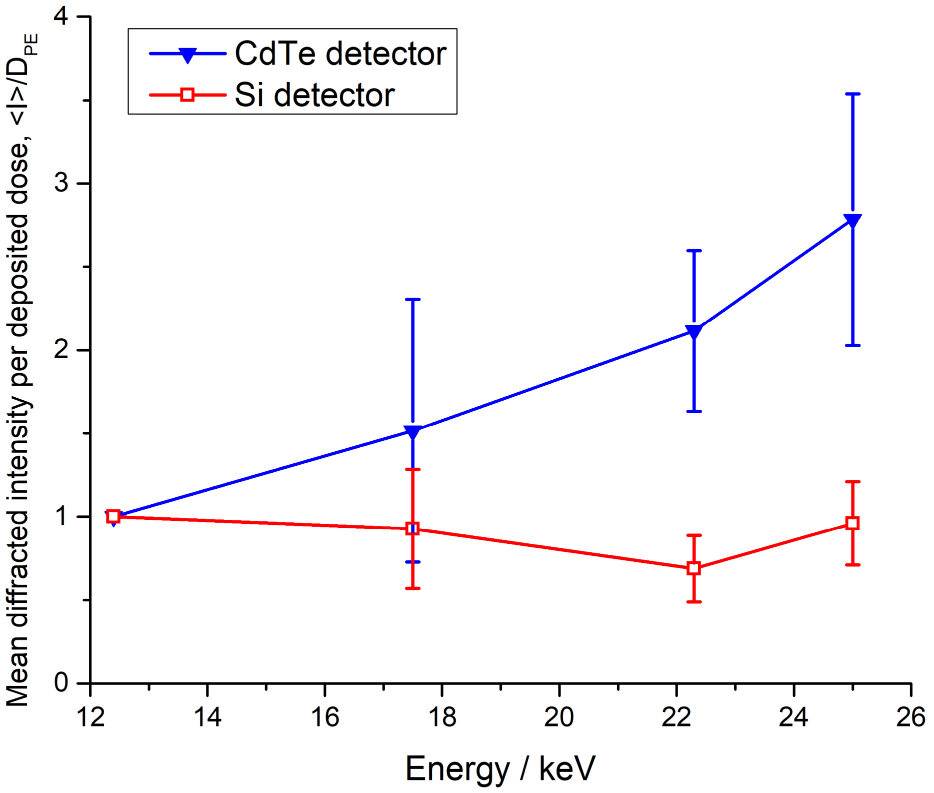

Figure S4 Increase in the diffracted intensity per unit deposited dose, <I>/D_{PE}, as a function of energy, normalised to 12.4 keV. Data shown are averaged from 29 data series (22 recorded using CdTe Eiger and 7 using the Si Pilatus) with the standard deviation at each energy shown as error bars. The mean diffracted intensity, <I>, is scaled to the illuminated crystal volume.

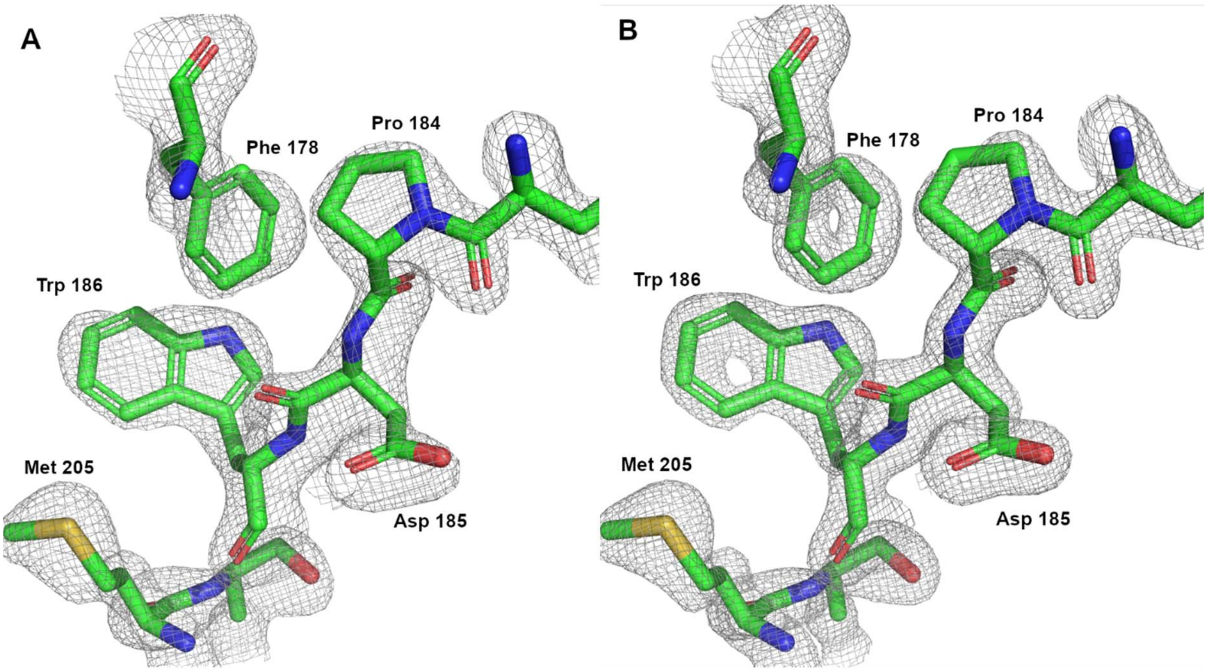

Figure S5 Composite omit maps illustrating differences between data recorded using the CdTe detector at different energies but the same total diffracted intensity. (A) Example volume of electron density obtained from 100 degrees of thermolysin data collected at 12.4 keV, diffracting to 1.90 Å using a $CC_{1/2}$>0.33 threshold. (B) electron density of the same volume obtained by collecting from the same position of the crystal at 25 keV and diffracting to 1.61 Å using the same criterion. The increased resolution is apparent in the electron density of rings and clarity of other features. Maps contoured to 1.5 σ in both cases. Models were refined and maps obtained using Phenix; R/R_{free} 0.210/0.245 in the case of (A) and R/R_{free} 0.202/0.229 in the case of (B). Figure created using Pymol (The PyMOL Molecular Graphics System, Version 2.0 Schrödinger, LLC).

Reference

D. Liebschner, P. V. Afonine, M. L. Baker, G. Bunkóczi, V. B. Chen, T. I. Croll, B. Hintze, L.-W. Hung, S. Jain, A. J. McCoy, N. W. Moriarty, R. D. Oeffner, B. K. Poon, M. G. Prisant, R. J. Read, J. S. Richardson, D. C. Richardson, M. D. Sammito, O. V. Sobolev, D. H. Stockwell, T. C. Terwilliger, A. G. Urzhumtsev, L. L. Videau, C. J. Williams, and P. D. Adams
Acta Cryst. (2019). D75, 861-877
